# Macular Microcysts in Mitochondrial Optic Neuropathies: Prevalence and Retinal Layer Thickness Measurements

**DOI:** 10.1371/journal.pone.0127906

**Published:** 2015-06-05

**Authors:** Michele Carbonelli, Chiara La Morgia, Giacomo Savini, Maria Lucia Cascavilla, Enrico Borrelli, Filipe Chicani, Carolina do V. F. Ramos, Solange R. Salomao, Vincenzo Parisi, Jerry Sebag, Francesco Bandello, Alfredo A. Sadun, Valerio Carelli, Piero Barboni

**Affiliations:** 1 IRCCS, Istituto delle Scienze Neurologiche di Bologna, Bellaria Hospital, Bologna, Italy; 2 Neurology Unit, Department of Biomedical and Neuromotor Sciences (DIBINEM), University of Bologna, Bologna, Italy; 3 G.B. Bietti Foundation, IRCCS, Rome, Italy; 4 San Raffaele Scientific Institute, Milan, Italy; 5 Department of Ophthalmology, Federal University of São Paulo, UNIFESP, São Paulo, Brazil; 6 Doheny Eye Institute, Los Angeles, University of California Los Angeles, Los Angeles, California, United States of America; 7 VMR Institute for Vitreous Macula Retina, Huntington Beach, California, United States of America; 8 Studio Oculistico d’Azeglio, Bologna, Italy; University of Houston, UNITED STATES

## Abstract

**Purpose:**

To investigate the thickness of the retinal layers and to assess the prevalence of macular microcysts (MM) in the inner nuclear layer (INL) of patients with mitochondrial optic neuropathies (MON).

**Methods:**

All patients with molecularly confirmed MON, i.e. Leber’s Hereditary Optic Neuropathy (LHON) and Dominant Optic Atrophy (DOA), referred between 2010 and 2012 were enrolled. Eight patients with MM were compared with two control groups: MON patients without MM matched by age, peripapillary retinal nerve fiber layer (RNFL) thickness, and visual acuity, as well as age-matched controls. Retinal segmentation was performed using specific Optical coherence tomography (OCT) software (Carl Zeiss Meditec). Macular segmentation thickness values of the three groups were compared by one-way analysis of variance with Bonferroni post hoc corrections.

**Results:**

MM were identified in 5/90 (5.6%) patients with LHON and 3/58 (5.2%) with DOA. The INL was thicker in patients with MON compared to controls regardless of the presence of MM [133.1±7μm vs 122.3±9μm in MM patients (p<0.01) and 128.5±8μm vs. 122.3±9μm in no-MM patients (p<0.05)], however the outer nuclear layer (ONL) was thicker in patients with MM (101.4±1mμ) compared to patients without MM [77.5±8mμ (p<0.001)] and controls [78.4±7mμ (p<0.001)]. ONL thickness did not significantly differ between patients without MM and controls.

**Conclusion:**

The prevalence of MM in MON is low (5-6%), but associated with ONL thickening. We speculate that in MON patients with MM, vitreo-retinal traction contributes to the thickening of ONL as well as to the production of cystic spaces.

## Introduction

Mitochondrial optic neuropathies (MON) are a group of hereditary diseases characterized by variable degrees of optic nerve atrophy, caused by mitochondrial dysfunction and consequent loss of retinal ganglion cells (RGCs) [1, 2]. The two most frequent non-syndromic forms of MON are Leber’s hereditary optic neuropathy (LHON) and dominant optic atrophy (DOA). LHON is a maternally inherited blinding disease due in 90–95% of cases to three primary mitochondrial DNA point mutations (11778/ND4, 3460/ND1 and 14484/ND6), affecting the mitochondrial respiratory complex I [3]. DOA is an autosomal dominant disorder due, in the large majority of cases, to heterozygous mutations in the OPA1 gene [4, 5]. The clinical expression of both LHON and DOA is characterized by the early and preferential involvement of the papillomacular bundle that often expands to a widespread loss of axons in the optic nerve, ultimately leading to severe optic atrophy [1, 2]. Consistent with this pattern of fiber loss, LHON and DOA patients experience central vision loss with central scotoma and dyschromatopsia, subacute for LHON and slowly progressive in DOA. These neurodegenerative disorders differ substantially in their pathogenic mechanism and clinical course from inflammatory optic neuritis as seen in multiple sclerosis (MS) or neuromyelitis optica (NMO) [6].

Optical coherence tomography (OCT) has become an essential tool for both diagnosis and follow-up of LHON and DOA patients. Previous studies carried out by time-domain OCT (TD-OCT) showed an increased thickness of the peripapillary retinal nerve fiber layer (RNFL) in the temporal quadrant in LHON carriers [7], a thicker RNFL in all quadrants except the temporal in patients with early LHON and a thinner RNFL in all quadrants in chronic LHON and DOA patients [8, 9]. OCT has also been extensively used in studying inflammatory optic neuritis, revealing that even in patients with MS without any episode of optic neuritis there is a measurable and progressive loss of axons and reduction of RNFL thickness [10].

Recently, spectral-domain OCT (SD-OCT) has been utilized for further characterizing the axonal loss and retinal features in both MON and inflammatory optic neuritis. In particular, SD-OCT investigations led to the recent observation of macular microcysts (MM) in inflammatory optic neuritis in MS and NMO, possibly correlating with the severity of the disease process [11–14]. It has been proposed that these changes may be useful in identifying the inflammatory nature of the pathology [11, 13]. However, in addition to patients with MS and NMO, MM have also been described in a case of compressive optic neuropathy [15], in optic neuritis not due to MS [16, 17], and in glaucoma [18]. We first noticed MM in LHON and DOA patients in 2009 (unpublished data) and recently discussed their nature, showing that SD-OCT could identify them in the inner nuclear layer (INL) of the macula in a small subgroup of MON patients in the absence of any leakage at the fluorescein angiography, thus adding other conditions in which MM may occur [19, 20].

In the present study, we used SD-OCT with macular segmentation to 1) assess the prevalence of MM in patients with LHON and DOA, 2) investigate the thickness of the different retinal layers in MON with and without MM, and 3) evaluate any difference between LHON and DOA patients with MM.

## Methods

### Study design and participants

We prospectively enrolled all patients with molecularly confirmed diagnoses of LHON (mtDNA pathogenic mutation) or DOA (OPA1 mutation) referred to the Unit of Neurology of the University of Bologna between 2010 and 2012. Ninety LHON patients and 58 DOA patients had an extensive ophthalmologic examination, including best-corrected visual acuity measurement, slit lamp biomicroscopy, intraocular pressure measurement, indirect ophthalmoscopy, optic nerve head photography and SD-OCT. Exclusion criteria were the presence of any retinal pathology and/or optic nerve disease other than LHON or DOA. We also excluded, in order to avoid confounding factors such as RNFL swelling, LHON patients in the acute phase (within two years from disease onset) or LHON patients with an atypically slowly progressive course.

Five LHON and three DOA patients with MM were identified (5.6% and 5.2% respectively). Due to the small sample size of the cohort both eyes of the eight patients with MM (13 eyes) were considered for the statitical analysis in comparison with the two control groups: 1) a sample of LHON and DOA patients without MM matched by age, RNFL thickness, visual acuity (36 patients), and 2) a control group (22 individuals). For the latter the inclusion criteria were the following: best corrected visual acuity above 1.0 decimals, normal appearance of the optic disc, normal visual field (the latter was examined with a SITA 24–2 standard test in all subjects using the Humphrey VF analyzer, HFA II 750–4.1 2005, Carl Zeiss Meditec Inc, USA), absence of significant ocular disease found by routine ophthalmological examination, absence of glaucoma history in the family and/or absence of systemic diseases with possible ocular involvement, such as diabetes mellitus. From this sample we randomly selected 22 eyes of 22 subjects matching the hereditary MON patients group for age, since this factor has been shown to influence peripapillary RNFL thickness, as measured by SD-OCT [21, 22]. Age has also been shown to influence the macular ganglion cell-inner plexiform layer (GCIPL) thickness [23]. In addition, inclusion criteria for both patients and controls were: refractive error between -3 and +3 diopters of sphere and between -2 and +2 diopters of cylinder, intraocular pressure less <21 mm Hg and absence of any other disease of the retina and/or optic nerve.

All participants gave their written informed consent for clinical and genetic investigations according to the Declaration of Helsinki and the study was approved by the internal review board at the University of Bologna (protocol number 123/2006/U/Sper).

### Procedures

All subjects underwent retinal nerve fiber layer (RNFL) thickness and ganglion cell layer-inner plexiform layer (GC-IPL) thickness measurements by SD-OCT (Cirrus HD-OCT, software version 6.0; Carl Zeiss Meditec, Inc, Dublin, CA, USA). All scans were acquired using the Optic Disc Cube 200x200 and the Macular Cube 512x128 protocols. After the patient had been properly seated and aligned, the iris was brought into view using the mouse-driven alignment system and the ophthalmoscopic image was focused. In order to acquire the Optic Disc Cube, the ONH was centered on the live image before the centering and enhancement was optimized. After the scanning process was launched, the instrument’s 840 nm wavelength laser beam generated a cube of data measuring 6 mm x 6 mm after scanning a series of 200 B-scans with 200 A-scans per B-scan (40,000 points) in 1.5 seconds (27,000 A-scans/sec). Cirrus HD-OCT algorithms found the optic disc and automatically placed a calculation circle of 3.46 mm diameter evenly around it. Layer-seeking algorithms found the RNFL inner (anterior) boundary and RNFL outer (posterior) boundary for the entire cube, excepting the optic disc. The system extracted from the data cube 256 A-scan samples along the path of the calculation circle. The resulting temporal, superior, nasal, inferior, temporal profile map was equivalent to the Stratus peripapillary RNFL (pRNFL) scan provided by TD-OCT (Stratus OCT, Carl Zeiss Meditec, Dublin, CA).

The ONH parameters reported resulted from a fully automatic algorithm that defined both the optic disc and cup margins within the three-dimensional data cube. The disc margin was defined as the termination of Bruch’s membrane (also referred to as “neural canal opening” or “Bruch’s membrane opening”) [24].

**Fig 1:**
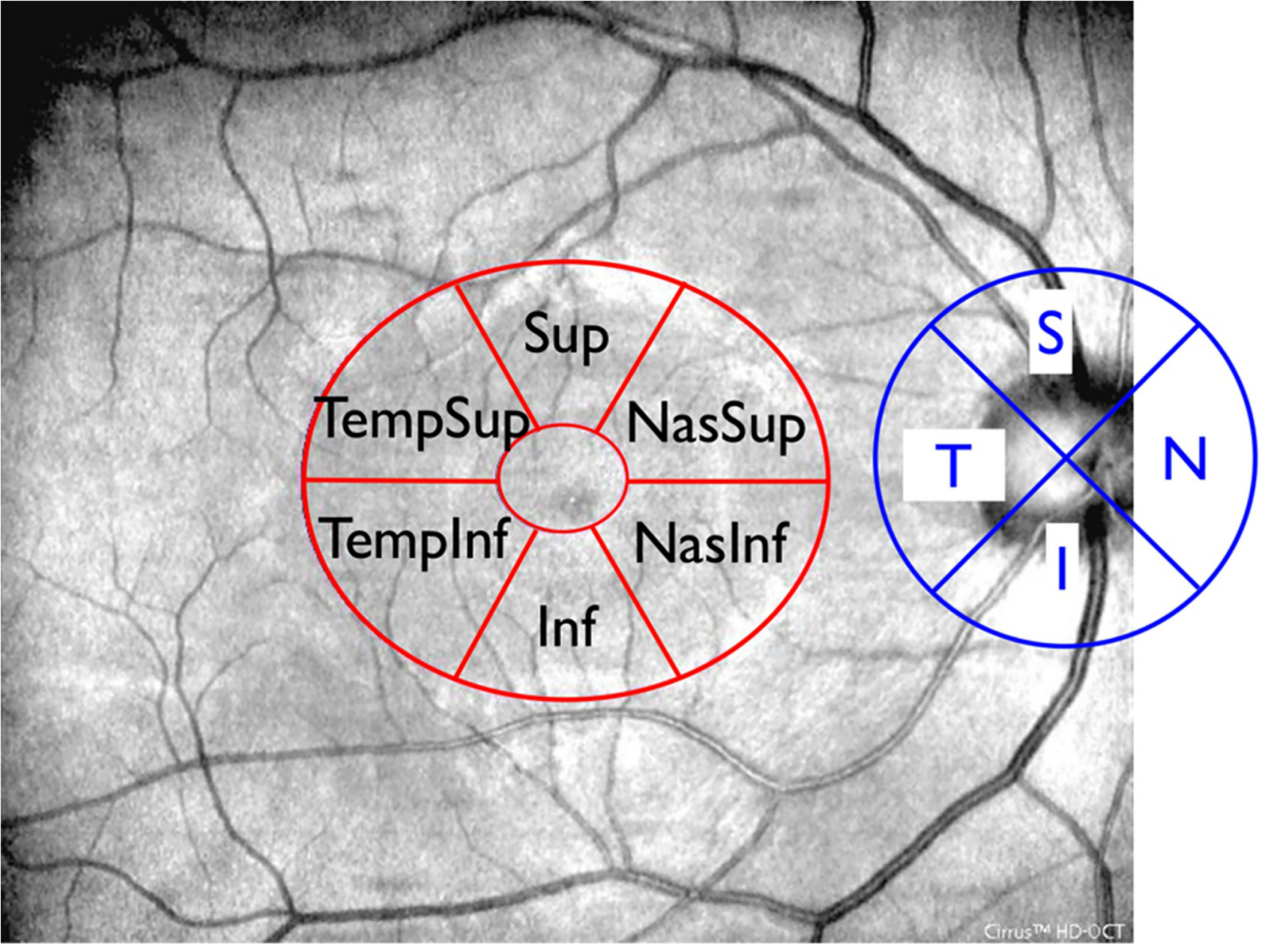
Superimposition of peripapillar RNFL quadrants and macular GC-IPL sectors to OCT fundus image. The four RNFL quadrants (temporal, superior, nasal, inferior) and the six GC-IPL sectoral (superotemporal, superior, superonasal, inferonasal, inferior, inferotemporal) thicknesses were measured from circular scan around the disc and from the elliptical annulus centered on the fovea respectively.

**Fig 2:**
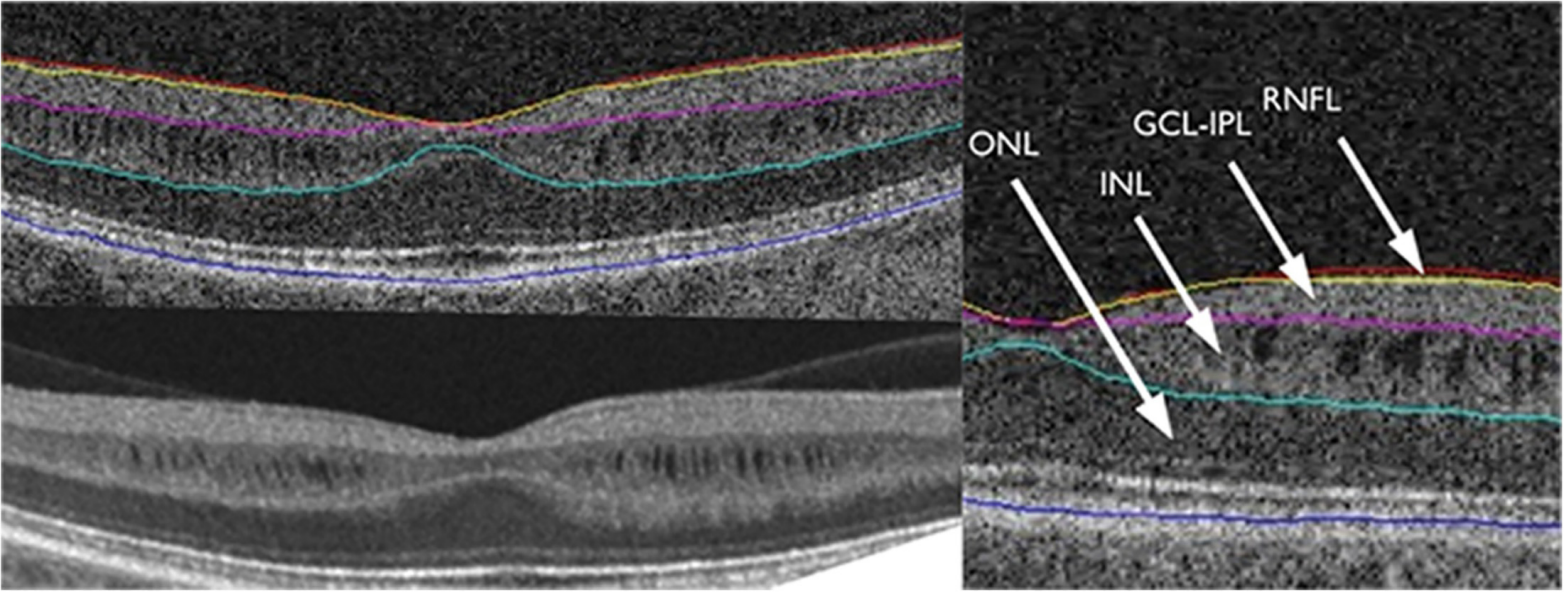
Sample OCT segmentation. Segmentation, by regular Cirrus algorithm, identifies the outer boundary of the macular RNFL (mRNFL), the outer boundary of the inner plexiform layer (IPL), and the outer boundary of the outer plexiform layer (OPL).

In order to acquire the Macular Cube, patients were asked to fixate on the central target. Macular data were analyzed by means of the Ganglion Cell Analysis (GCA) algorithm, which detected and measured the thickness of the macular ganglion cell-inner plexiform layer (GC-IPL) within a 14.13-mm^2^ elliptical annulus area centered on the fovea (Fig 1). The size and shape of the annulus were chosen because this conforms more closely to the real anatomy, and this annulus corresponds to the area where the RGC layer is thickest in normal eyes. The average, minimum, and six sectoral (superotemporal, superior, superonasal, inferonasal, inferior, inferotemporal) GC-IPL thicknesses were measured from the elliptical annulus. A detailed description of how the algorithm operates has been presented in detail [23]. Retinal segmentation was carried out according to the technique described by Saidha et al. using specific software provided by Carl Zeiss Meditec (Fig 2) [13]. Segmentation undertaken in three dimensions identified the outer boundary of the macular RNFL (mRNFL), the outer boundary of the inner plexiform layer (IPL), and the outer boundary of the outer plexiform layer (OPL) (Fig 2). It could therefore yield the thicknesses of the following macular layers: the mRNFL, GCL and inner plexiform layer (GCL-IPL, given by the difference between the mRNFL and the IPL segmentation), inner nuclear layer (INL) including the outer plexiform layer (given by the difference between the IPL and OPL segmentations), and outer nuclear layer (ONL) including inner and outer photoreceptor segments (given by the difference between the OPL and the retinal pigment epithelium segmentation identified by regular Cirrus algorithm).

Only high-quality scans, defined as scans with signal strength ≥ 7, without RNFL discontinuity or misalignment, involuntary saccadic or blinking artifacts, and absence of algorithm segmentation failure on careful visual inspection, were used for analysis.

### Statistical analysis

The measurements of the macular segmentation thickness were compared among the 3 groups by one-way analysis of variance (ANOVA). For post-hoc pairwise comparisons between groups Bonferroni correction was applied. The means, standard deviation (SD) of means, and Pearson’s correlation coefficient were calculated. All statistical analyses were performed using GraphPad InStat 3 for Macintosh and p value <0.05 was considered statistically significant.

## Results

### Prevalence of MM and demographic data of the investigated samples

We investigated 90 LHON and 58 DOA patients. 81 LHON patients carried one of the three common mtDNA point mutations (11778/ND4, 3460/ND1 and 14484/ND6) and 9 carried rare mtDNA mutations (3733/ND1, 14482/ND6, 3890/ND1 and 14495/ND6) [25, 26]. All DOA patients had a molecularly confirmed OPA1 mutation.

**Fig 3:**
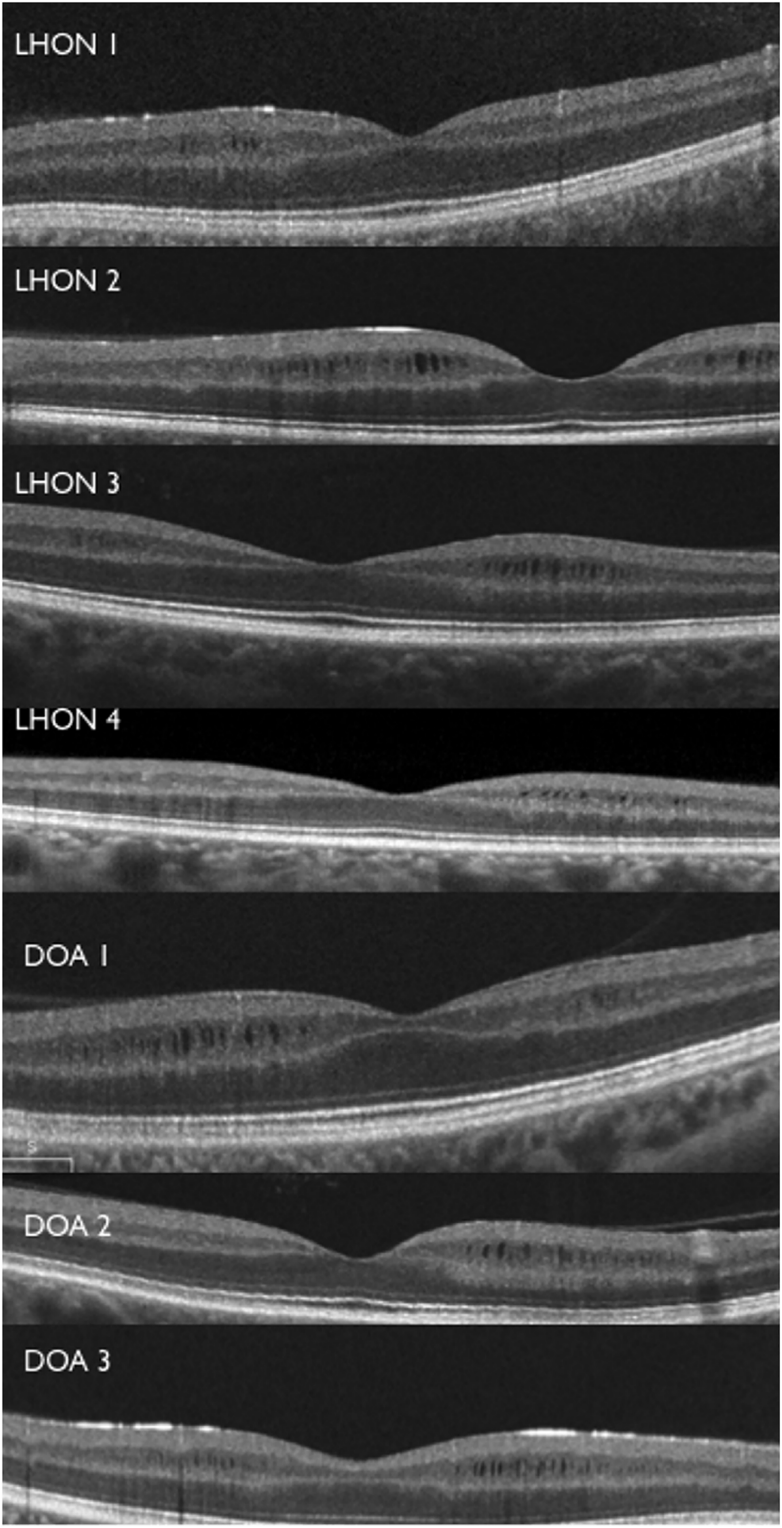
Patients OCT foveal scans. Horizontal OCT scans revealed macular microcysts (MM) of the inner nuclear layer in the four LHON patients and three DOA patients.

Macular microcysts were identified in 5 patients out of 90 with LHON (5.6%) and 3 patients out of 58 with DOA (5.2%) (Fig 3). Amongst LHON patients, 2 patients carried the 11778/ND4, 1 patient the 3460/ND1, 1 patient the 14484/ND6 and 1 patient the 14459/ND6 mutation. For DOA patients with OPA1 mutation, 1 patient carried 2708_11 delTTAG in exon 27, 1 patient c.2196_2197 dup AGAC in exon 22, and 1 patient c.1409A>G in exon 14. One LHON patient with MM was excluded from the study because of motion artifacts that did not allow the correct macular segmentation.

The control group of MON without MM was composed by 16 LHON patients who carried the 11778/ND4 (14 patients), 3460/ND1 (1 patient) and 3890/ND1 (1 patient) mtDNA mutations and 20 DOA patients, who carried an OPA1 mutation.

Table 1 shows the demographic data of LHON and DOA patients with and without MM, and age-matched controls.

**Table 1:** Demographic, clinical characteristics of the study participants.

	LHON		DOA		Healthy Controls
	MM	No-MM	MM	No-MM	No-MM
*Number of participants (number of eyes)*	4 (7)	16 (16)	3 (6)	20 (20)	22 (22)
*Age*, *years*	30±6	31±9	35±13	37±16	34±12
*Visual acuity*, *decimals*	0.07±0.03	0.12±0.10	0.28±0.22	0.39±0.24	1.25
*360° pRNFL*, *mμ*	60±6	60±7	67±17	66±4	93±10

Data are mean (SD). LHON = Leber hereditary optic neuropathy. DOA = Dominant optic atrophy. pRNFL = peripapillary retinal nerve fiber layer. MM = macular microcysts.

### Macular segmentation in MON patients with and without MM vs control group

ANOVA showed a statistically significant difference for all the examined parameters in the three groups (p<0.0001 for all variables except INL thickness with p = 0.0009).

We found that total macular thickness (p<0.05 and p<0.001 respectively), mRNFL (p<0.001 both groups) and GCL-IPL thickness (p<0.001 both groups) were significantly reduced in both groups of MON patients compared with controls (Table 2). Total macular thickness and GCL-IPL were significantly higher in MON patients with MM compared to those without MM (p<0.001), whereas the mRNFL thickness was not significantly different between the two groups. The INL was thicker in MON patients with and without MM compared to healthy controls (p<0.01 and p<0.05 respectively) but there was no significant difference between patients with MM and without MM, probably due to the small sample size. Also the analysis of each perifoveal sector failed to reveal any difference in terms of INL thickness between MON patients with and without MM. The ONL was thicker in MON patients with MM compared to both MON patients without MM (p<0.001) and controls (p<0.001), whereas no significant differences were detected in the ONL layers between MON patients without MM and healthy controls (small sample size).

**Table 2:** Comparison of optical coherence tomography parameters (different macular thickness layers) between all patients with and without macular microcysts and healthy controls.

*Macular Thickness Average (mμ)*	MM	no-MM	Healthy Controls	MM vs controls	MM vs No-MM	No-MM vs controls	Rate of change MM Vs controls (%)	Rate of change no-MM Vs controls (%)
*Total thickness*	305.1±2	271.3±1	319.3±1	<0.05	<0.001	<0.001	-4.4	-15
*mRNFL*	13±2	14.3±4	34.6±3	<0.001	ns	<0.001	-62.4	-58.6
*GCL-IPL*	57.4±6	50.9±4	83.9±6	<0.001	<0.001	<0.001	-31.5	-39.3
*INL*	133.1±7	128.5±8	122.3±9	<0.01	ns	<0.05	8.8	5
*ONL*	101.4±1	77.5±8	78.4±7	<0.001	<0.001	ns	29.3	-1

Data are mean (SD). LHON = Leber hereditary optic neuropathy. DOA = Dominant optic atrophy. MM = macular microcysts. mRNFL = macular retinal nerve fiber layer. GCL-IPL = ganglion cell and inner plexiform layers. INL = inner nuclear layer including outer plexiform layers. ONL = outer nuclear layer including inner and outer photoreceptor segments.

Each patient with MM showed the typical pattern previously reported: six patients with the perifoveal crescent-shape distribution of the MM and five patients with the perifoveal oval-shape distribution more extensive in the nasal sector [17–20].

### Comparison between LHON and DOA patients

Table 3 separately compares the INL and ONL of LHON and DOA patients with and without MM to controls.

**Table 3:** Comparison of optical coherence tomography parameters (different macular thickness layers) between LHON and DOA patients with and without macular microcysts and healthy controls.

*Macular Thickness Average (mμ)*				p value	p value	p value
	LHON-MM	LHON no-MM	Healthy Controls	LHON-MM vs controls	LHON-MM vs LHON-no-MM	LHON no-MM vs controls
*INL*	131.8±7	127.6±8	122.3±9	<0.05	ns	>0.05
*ONL*	105.0±2	81.0±6	78.4±7	<0.001	<0.001	>0.05
	DOA-MM	DOA no-MM	Healthy Controls	DOA-MM vs controls	DOA-MM vs DOA no-MM	DOA no-MM vs controls
*INL*	134.6±7	129.3±9	122.3±9	<0.01	ns	<0.05
*ONL*	97.3±8	74.7±8	78.4±7	<0.001	<0.001	ns

Data are mean (SD). MM = macular microcysts. INL = inner nuclear layer including outer plexiform layers. ONL = outer nuclear layer including inner and outer photoreceptor segments.

INL thickness was increased in LHON with MM and DOA patients with and without MM compared to controls (p<0.05 for LHON; p<0.01 and p<0.05 for DOA with and without MM respectively), whereas there was no significant difference between LHON patients without MM and controls. The INL thickness did not differ between LHON and DOA patients.

ONL was significantly thicker in LHON and DOA patients with MM compared to both MON patients without MM and controls (p<0.001 for all groups), whereas ONL thickness was not significantly different between MON patients without MM and healthy controls.

Furthermore, ONL thickness did not differ between LHON and DOA patients with MM.

## Discussion

The present study demonstrates that MM occur in about 5–6% of patients with MON. This finding strikingly differs from a report by Wolff et al. [27] in which MM were detected in 30 cases (75%) of optic atrophy due to mitochondrial dysfunction, but it is in close agreement with previous studies on DOA patients reporting that only a minor fraction (about 4%) of DOA patients has MM [28]. Detailed OCT retinal segmentation of the LHON and DOA patients compared with a control population shows that the innermost retinal layers (mRNFL, GCL-IPL) are thinner in the MON patients, whereas the INL appears thicker in the MON patients (with or without MM) and ONL is significantly thicker only in the MM subgroup of MON patients. The most likely explanation for these results is that the RGC loss directly reflects on the RNFL and GCC, which become thinner. If the retina is held by firm vitreo-retinal adhesion then the underlying retina must either expand or split to form empty cystic spaces (MM).

The total macular thickness is significantly thinner in patients without MM compared with both patients with MM and controls. Our finding that the nuclear retinal layers (INL and ONL) of patients with MON exhibit an increase in thickness compared to controls is of particularly interest. We postulate that, in conjunction with a reduction of RNFL and GCL-IPL thicknesses, the retina is probably subjected to a negative pressure that limits further thinning and leads to an increased thickness of the INL, the occasional formation of MM and a greater thickening of the ONL in a subgroup of patients.

The greater percentage of increase in the thickness of the ONL compared with the INL (29% vs 8.8% in patients with MM) and the lack of cyst formation in this layer might represent a different elastic coefficient reflecting the different cell populations and architecture of these layers. It is well-known that the compressive modulus of the choroid and RPE combined (a loose vascular connective tissue and neuro-epithelial monolayer) is more than 40% lower than that of the retina as a whole and this may affect the elastic response of the outer retinal layers connected with RPE and subjected to vitreous adhesion or traction [29].

The force with the above-mentioned characteristics, which might be a factor for these changes, is posterior vitreo-macular traction (VMT) due to persistent vitreo-macular adhesion and likely vitreo-papillary adhesion. All eyes with MM, in fact, showed persistent adherence of posterior vitreous cortex to the macula. In this scenario, the retina cannot collapse if its anterior aspect is held fixed by the vitreous, so intra-retinal dehiscences occur in consequence to the negative pressure and MM may arise. Thus, in a similar fashion to macular holes, cysts are not exudative, but tractional, as expected for non-inflammatory conditions such as LHON and DOA. In eyes with lamellar macular holes and macular pucker, persistent vitreo-papillary adhesion alters the vectors of force at the macula inducing tractional MM [30–34]. We propose that a similar mechanism occurs in the MON-associated cases of optic atrophy described herein.

Reduction of the RNFL thickness also occurs in inflammatory optic neuritis and MM have been reported in these conditions and, in fact, proposed as a specific indicator of disease and severity [11–13, 35]. The present study, as well as other studies, show that MM are therefore likely to be a non-specific byproduct of optic atrophy, which becomes manifest only in a subgroup of predisposed patients [16, 18–20, 27, 28]. We do not believe that MM are a biomarker specific of inflammatory diseases of the optic nerve and retina, and they should not be used as a marker for disease severity.

The MM patients here reported were all in their third decade, pointing to a possible role of young age in favoring the MM formation, as already suggested by Abegg et al [36]. We propose that the relatively young age of these patients supports the hypothesis of vitreo-retinal adhesion, as vitreous is known to be more firmly adherent in youth, especially at the macular level [37]. This firm adhesion at the macula produces a vector opposite to that generated by macular thinning. It is not surprising, therefore, that MM are primarily seen in young-adults with severe optic atrophy, such as MS and NMO, as well as in LHON and DOA.

It has also been proposed that MM are caused by the effects of trans-synaptic retrograde degeneration [15, 36]. However, the latter was demonstrated to cause atrophic thinning of the INL [38]. In contrast, our study demonstrated a thickening of INL. A possible explanation might be that INL trans-synaptic retrograde atrophy induces a structural retinal weakening, which, in conjunction with the tractional force of the vitreous, leads to the observed thickening, and occasionally to MM. Furthermore, Abegg et al. [36] reported a patient who exhibited a decrease of MM in the course of a longitudinal observation period that cannot be explained by any reversal of trans-synaptic degeneration, but might be due to release of vitreo-macular adhesion from age-related changes.

Another notable finding is the topographic localization of the MM in the macula of these patients. Previous studies have shown that the posterior vitreous cortex overlying the macula is thinned in a disc-shaped area centered at the fovea that is about 4 disc diameters in size [39–41]. It is thought that the arcuate edge of this disc-shaped zone represents a site of increased vitreo-retinal adhesion and a source of abnormal vitreo-macular traction. This zone appears to correspond quite closely with the distribution of MM in the patients described here, further supporting the hypothesis of vitreo-macular adhesion/traction as a factor underlying the pathophysiology of MM.

All considered, the hypothesis of vitreous traction as major force generating MM, is a likely explanation, which does not exclude the co-participation of other factors including trans-synaptic retrograde degeneration or other unknown mechanisms that may all act synergistically in a small subset of patients. Future studies are warranted, in particular including a larger sample size of patients with MM. This would allow to avoid the potential limitation of the within-subject inter-eye correlation due to inclusion of both eyes as in the current study. Moreover, it would be useful to use ultrasonography for evaluation of posterior vitreous detachment, as well as OCT imaging of the vitreo-papillary interface to carefully assess the role of vitreous in the pathogenesis of MM in these and other optic nerve disorders.
